# Bonding with nature: a validation of the dispositional empathy with nature scale in Italy

**DOI:** 10.3389/fpsyg.2025.1388798

**Published:** 2025-02-28

**Authors:** Chiara Lovati, Federico Manzi, Cinzia Di Dio, Davide Massaro, Gabriella Gilli, Antonella Marchetti

**Affiliations:** ^1^Research Unit on the Psychology of Arts and Environments, Department of Psychology, Università Cattolica del Sacro Cuore, Milan, Italy; ^2^Research Center on Theory of Mind and Social Competence in the Lifespan (CeRiToM), Department of Psychology, Università Cattolica del Sacro Cuore, Milan, Italy; ^3^Research Unit on Theory of Mind (UniToM), Department of Psychology, Università Cattolica del Sacro Cuore, Milan, Italy; ^4^Research Unit in Robotics and Psychology in the Lifespan (PsyRoLife), Department of Psychology, Università Cattolica del Sacro Cuore, Milan, Italy; ^5^IRCCS Don Carlo Gnocchi Onlus Foundation, Milan, Italy

**Keywords:** dispositional empathy with nature scale, DEN, Italian validation, environment, pro-environmental behaviors

## Abstract

This study proposes a psychometric validation of the Italian version of the Dispositional Empathy with Nature (DEN). Scientific research data has found high levels of environmental concern among people around the world, showing that majority of the population is aware of the seriousness of the environmental problems we are witnessing, as well as is conscious of the damage that some of their behaviors cause to the environment. Based on this premise, Empathy with Nature could be an important educational strategy for addressing the environmental crisis. A study was conducted involving 307 Italian adults (CFA = 146; 45.9% women; 54.1% man; Mean age = 34.65; *SD* = 11.770); (EFA = 161; 50.3% women; 49.7% man; Mean age = 34.30; *SD* = 10.360) to o assess the psychometric properties of a scale in the Italian context. The study aimed to establish the internal consistency of the DEN scale and evaluate its convergent, discriminant, and predictive validity. Both confirmatory and exploratory factor analyses, using a split sample, supported the one-factor structure consistent with the original version proposed by Tam. These findings strongly suggest that the DEN scale is reliable and valid in the Italian context.

## 1 Introduction

The term empathy was used in ancient Greece to indicate the emotional relationship of participation that bound the author-songwriter (*aedo*) to her/his audience. Empathy, from Greek “*en-pathos*” (“to feel within”), consists of recognizing the emotions of others as one’s own, allowing the understanding of others’ views, thoughts, feelings, and emotions (Sunassee et al., 2021). The term empathy was coined by Robert Vischer, a scholar of figurative arts and esthetics, at the end of the 19th century (Freedberg and Gallese, 2007). This term originated within a context of esthetic reflection, where empathy refers to the capacity of the human imagination to grasp the symbolic value of nature. Vischer conceptualized this idea as the ability to internally sense and connect with external stimuli, perceiving nature as part of our own being. Therefore, it signifies the capability to extend emotions from us to others and to objects (Rifkin, 2010). At the beginning of the 20th century, Lipps introduced the construct of empathy in psychology, speaking of profound participation in the experience of another human being (Freedberg and Gallese, 2007). Empathy is a complex psychological process that allows people to understand the experiences of others “as if they were their own” while at the same time keeping them distinct from their own (Kunyk and Olson, 2001). According to one of the most widely accepted theories, empathy is a process based on an embodied simulation, which is essentially motor experience characterized by neurons that would act immediately prior to more properly cognitive processing, i.e., mirror neurons (Rizzolatti, 2005; Rizzolatti and Craighero, 2005; Gallese et al., 2006; Penagos-Corzo et al., 2022; Peng et al., 2024). They are called ‘mirror neurons’ and, specifically, they are selectively activated either when a certain type of transitive action is performed or when someone is seen performing the same type of action. It is interesting that the activation of mirror neurons is independent of the subject being observed performing the action (Gallese et al., 2006). The sharing of the same bodily state between two people enables this direct form of understanding of the other’s experience, which has been termed empathic (Gallese et al., 2006). The intersubjective role of mirror functions has been investigated, demonstrating their involvement in motor skill learning and imitative processes (Rizzolatti and Craighero, 2005). The first and most fundamental tool human beings have for understanding the other’s intention is to “put ourselves in their shoes,” empathically transposing ourselves into the concreteness of their experience. By “simulation” is therefore meant the process of understanding the other’s world through attending to their intentional state (attitudes, actions, emotions, etc.). Thus, embodied simulation skips the higher capacities of intellectual understanding, focusing rather on the relationship between the body and the understanding of the external world that is the basis of empathy (Rizzolatti, 2005; Rizzolatti and Craighero, 2005; Gallese et al., 2006; Gallese, 2009; Bekkali et al., 2021; Simon and Gutsell, 2021; Feng et al., 2022; Penagos-Corzo et al., 2022; Plata-Bello et al., 2023).

Humans establish a conniving relationship with their environment, being able to create a relationship of empathy with all their surroundings such as nature, animals and ecosystem (Hall and Schwartz, 2019). While empathy between humans has a long tradition in empirical research in the humanities and social sciences (Gazzola et al., 2006; Decety, 2010; Harrison and Hall, 2010), empathy toward nature request further investigation (Tam, 2013; Brown et al., 2019). In this context, Brown et al. (2019) argued that empathy toward nature involves emotionally understanding the events within the natural world. In other words, observing a natural environment being mistreated (e.g., polluting it) generates a sense of pain/displeasure in people, i.e., empathic mirroring (Verplaetse et al., 2009; Lumber et al., 2017). From a theoretical perspective, empathy is considered to consist of both emotional and cognitive aspects (Sevillano et al., 2007). Emotional empathy involves experiencing the feelings of others, while the cognitive dimension involves understanding those emotions. Similarly, empathy toward nature involves the ability to empathize with the emotional states of the natural world, such as recognizing the distress of an animal affected by habitat pollution or observing the gradual decline of a natural environment (Tam, 2013). In recent decades, scientific research in the field of cognitive neuroscience has unveiled surprising connections between our experience of nature and our capacity for empathy toward other living beings. It has been demonstrated that observing nature activates several areas in our brain that are involved in the empathic experience (Sevillano et al., 2007; Fido and Richardson, 2019; Blythe et al., 2021; Wang et al., 2022).

When immersed in nature, we are not merely passive observers of the surrounding landscape, but we experience it actively and deeply. For example, It has been revealed that exposure to nature stimulates the same brain areas involved in the experience of empathy toward other human beings (Jing et al., 2022). This link between natural experience and empathy has implications for our understanding of the world and for our relationship with the environment that surrounds us. In an era where our relationship with the natural environment has become increasingly distant and fragmented, these findings highlight the urgent need to cultivate a deeper, more meaningful connection with nature. They invite us to rethink our relationship with the natural world—not as one of dominance and exploitation, but as an empathetic bond that unites us with all forms of life sharing our planet (Gilli et al., 2022; Jami et al., 2024).

Related to this, general nature connections and knowledge-based activities are often used to engage people with nature in order to comprehend how to protect it (Massaro et al., 2012; Di Dio et al., 2016; Lumber et al., 2017; Gilli et al., 2022). The increasing alienation of humans from nature has spurred researchers to examine this relationship as a method of restoring the environment. In recent years, several studies investigated the association between connection to nature and empathy concerning the natural environment (Cheng and Monroe, 2012; Brymer et al., 2019; Nilsson et al., 2019; Baceviciene and Jankauskiene, 2022; Jing et al., 2022). Reconnecting with nature refers to the inclination to foster collaboration and mutual intimacy with the natural world and other living beings, leading to beneficial outcomes for both personal well-being and environmental stewardship (Brown et al., 2019). Fido and Richardson (2019) have shown that connectedness to nature and empathy toward the natural world stimulates people to take care of and respect the environment. In fact, it is necessary to engage the emotional sphere, to consider the feelings that emerge from contact with nature and the degree of affiliation we have with it (Hughes et al., 2019). In this context, the motivational factors underlying a sustainable lifestyle have been analyzed in recent years. In general, feeling connected to something or someone does and feeling empathy with it triggers protective and altruistic behaviors toward that person. This reflection suggests that we should complement a knowledge of the environment to be protected with an understanding of the reasons for preserving it. In the light of this foundation, research findings indicate that re-establishing connections between humans and natural environments could serve as a crucial educational strategy for tackling the environmental crisis (Schultz, 2002; Davis et al., 2009; Andersson et al., 2014; Frantz and Mayer, 2014; Seymour, 2016; Ives et al., 2018). There is growing evidence that empathy can be used for environmental education and proximity to the environment and that empathy may be a powerful capacity for creating more responsible environmental behavior (Berenguer, 2007; Tam, 2013; Young et al., 2018); The strong correlation found between empathy and nature has motivated researchers and conservationists to introduce a new psychological concept: “empathy with nature” (Tam, 2013). This concept involves understanding and sharing the emotional aspects of the natural world. People who are in a relationship with nature also learn to feel emotionally close to the environment and consequently to defend it in case of threat (Perrin and Benassi, 2009; Lumber et al., 2017). That is one of the reasons why, nowadays, discussing sustainability and ecology should start from recovering the relationship of affiliation and affection with the natural world and the environment. A growing consensus suggests that individuals in Western countries need to modify their behavioral patterns (Fischer et al., 2012; Frantz and Mayer, 2014; Abson et al., 2017). A pioneering study by Berenguer (2007) showed that the more people experiencing empathy toward nature, such as a suffering animal or plant, the greater were the pro-environmental behavior and caring attitudes toward nature. These findings support the idea that empathy is an important psychological process to induce concrete behavioral changes toward nature and, consequently, a basis for designing effective environmental education interventions.

The development of validated and reliable tools to investigate dispositional empathy with nature and its connections to psychological well-being, as well as pro-environmental attitudes and behaviors, is increasingly warranted in the Italian context, as indicated by the growing interest in this dimension (Morandini, 2020; Oliva, 2022). Environmental and climate issues are now an increasingly recurring theme in Italian social and political debate. This growing interest by the society and institutions underlines the urgency of finding new approaches to environmental issues, and empathy toward the natural world seems to be a particularly interesting direction.

The present study aims to validate the Dispositional Empathy with Nature Scale (DEN; Tam, 2013) measuring human’s affective, cognitive and empathy with nature. To effectively address environmental challenges, it is essential for individuals to comprehend and resonate with the emotional essence of the natural world, as this correlates with a heightened inclination toward pro-environmental actions. We are interested in whether the DEN maintains comparable psychometric properties to its English version, thereby aiding in the evaluation of the empathy-with-nature construct. Additional validations of the DEN can be found in the available literature; for instance, Sevillano et al. (2007) conducted a first validation study in the Spanish context, demonstrating remarkable reliability of the instrument. We assume that the Italian adaptation will reveal a similar one-factor structure to the original version. Thus, our aim is to tailor and validate the DEN specifically for the Italian context, thereby enriching the exploration of the connection between human behaviors and the natural world. To achieve this objective, we conducted a study to provide evidence on the internal consistency, convergence, discriminant and predictive validity of DEN, with the aim of offering new cultural perspectives and stimulating further investigation into the relationship between empathy, nature, attitudes and behaviors of Italians toward the environment.

## 2 Aims

Considering the increasing attention to human-nature interaction and the need to evaluate it, our specific objectives were to:

- Examine the factorial validity of the Italian translation of the Dispositional Empathy with Nature Scale through confirmatory factor analysis (CFA) and exploratory factor analysis (EFA). The factorial validity of the DEN was examined according to the framework proposed by Tam (2013). We applied Hu and Bentler’s (1999) criteria across different fit indices to assess the model’s compatibility with the data. Our hypothesis was that the Italian version of the DEN would maintain a single-factor structure like the original version of the scale.
- Assess the reliability of the DEN and its concurrent, convergent, and divergent validity by examining its associations with psychological constructs related to the empathy toward nature. Specifically, these constructs included connection to nature, measured by the Connectedness to Nature Scale; moral disengagement from nature, assessed using the Civilian Moral Disengagement Questionnaire; and pro-environmental behavior, evaluated through the Pro-Environmental Behavior Scale Questionnaire (for details of the scales used see the Procedures and Methods section).

Our hypotheses for each type of validity are as follows:

- Convergent validity: We hypothesize that DEN scores will positively correlate with the Connectedness to Nature Scale, as both constructs share a similar psychological foundation: a deep personal bond with nature.
- Divergent validity: We anticipate a negative correlation between DEN scores and moral disengagement from nature. This is because moral disengagement represents an opposing construct to the sense of environmental duty and connection that underpin the DEN.
- Predictive validity: We hypothesize that individuals with a stronger connection to nature will demonstrate heightened environmental consciousness and responsibility, which is reflected in more substantial pro-environmental behavior.
- For divergent validity, we expect a negative correlation between DEN scores and moral disengagement from nature, as higher moral disengagement is theoretically opposite to the sense of environmental duty and connection inherent in the DEN construct.

## 3 Method

### 3.1 Participants

The sample included 307 Italian adults (CFA = 146; 45.9% women; 54.1% man; Mean age = 34.65; *SD* = 11.770) (EFA = 161; 50.3% women; 49.7% man; Mean age = 34.30; *SD* = 10.360). The sample size was determined following the criteria outlined by Austin and Steyerberg (2015). Table 1 provides an overview of the sociodemographic characteristics of the samples. Prolific’s data quality policies, which include rigorous participant verification, helped ensure the reliability of responses by maintaining high standards for data integrity. Every participant was rewarded with £2.25 per 15 min. All participants provided written informed consent after receiving a comprehensive explanation of the study procedure. The experimental protocol of the study received approval from the local Ethics Committee of the Department of Psychology at the niversità Cattolica del Sacro Cuore Milan, Italy.

**Table 1:** Socio-demographic characteristics of the exploratory and confirmatory factor analysis sample.

Sociodemographic characteristics	Construction sample EFA *N* = 146	Construction sample CFA *N* = 161
Age, mean ± SD	34.65 ± 11.770	34.30 ± 10.360
**Gender**	*N* (%)	*N* (%)
Man	67 (45.9%)	80 (49.7%)
Women	79 (54.1%)	81 (50.3%)
**Residence**		
North Italy	79 (50%)	70 (43.5%)
Center Italy	27 (18.5%)	29 (18%)
South Italy	28 (19.2%)	42 (26.1%)
Sicily and Sardinia	18 (12.3%)	20 (12.4%)
Outside Italy	–	–
**Educational level**		
Middle school or below	48 (32.9%)	65 (40.3%)
High school	38 (26%)	40 (24.8%)
Graduate school	44 (30.1%)	44 (27.3%)
Postgraduate school	14 (9.6%)	10 (6.2%)
Other	2 (1.4%)	2 (1.2%)
**Employment status**		
Student	42 (28.8%)	37 (23%)
Workman	6 (4.1%)	4 (2.5%)
Employed	55 (37.7%)	71 (44.1%)
Freelance	25 (17.1%)	24 (14.9%)
Unemployed	7 (4.8%)	18 (11.2%)
Pensioner	2 (1.4%)	1 (0.6%)
Other	9 (6.2%)	6 (3.7%)

The research was overseen and approved by the Ethics Committee for Research in Psychology (CERPS). The protocol number assigned to this research is 100-23.

### 3.2 Procedure and measures

The DEN scale items were back-translated following the standard guidelines (see Herdman et al., 1998; Beaton et al., 2000; Wild et al., 2005). More specifically, the questionnaire underwent the translation process, including initial translation by a native speaker of the target language, back-translation by an independent native speaker proficient in the original language, and a comparative review to ensure consistency and clarity. No discrepancies were identified, and the final version was refined accordingly. Data collection was conducted via an online survey. Data collection was conducted via an online survey administered on the Qualtrics platform. Initially, participants provided sociodemographic information, including age, gender, residence, occupation, and level of study. Subsequently, they completed four questionnaires randomly: Dispositional Empathy with Nature Scale, Connectedness to Nature Scale, Civic Moral Disengagement and Pro-Environmental Behaviors Scale. These scales are explained in detail in the following paragraphs.

#### 3.2.1 Dispositional empathy with nature

The Dispositional Empathy with Nature (DEN; Tam, 2013) consists of 10 items rated on a seven-point Likert scale, ranging from “strongly disagree” to “strongly agree.” It assesses the dispositional inclination to comprehend and empathize with the emotional essence of the natural environment. Specifically, DEN aims to investigate an individual’s affective and experiential empathy with nature, including understanding and sharing empathy in contact with the natural world. High internal reliability was demonstrated in the validation sample (α = 0.90). Here are some examples of the items: item1 “I imagine how I would feel if I were one of the suffering animals and plants”; item 2 “I get emotionally involved with the feelings of suffering animals and plants”; item 7 “I feel tenderness and concern for suffering animals and plants.”

#### 3.2.2 Connectedness to nature scale

The Connectedness to Nature Scale (CNS; Mayer and Frantz, 2004; and validated in Italian by Lovati et al. (2023)) consists of 14 items rated on a five-point Likert scale, ranging from “strongly disagree” to “strongly agree.” This scale evaluates individuals’ emotional bond with nature, commonly used in social and environmental psychology (e.g., item 1 “I often feel a sense of oneness with the natural world around me”; item 3 “I recognize and appreciate the intelligence of other living organisms”; item 8 “I have a deep awareness of how my actions affect the natural world”). Assesses how deeply individuals feel connected to the natural world, emphasizing emotional and experiential aspects. The concept of nature connection reflects the intricate relationship between people and their surroundings. In validation studies, the CNS demonstrated strong internal consistency, with a Cronbach’s alpha coefficient of 0.84 in the sample.

#### 3.2.3 Civilian moral disengagement

The Civilian Moral Disengagement (DMC) scale developed by Caprara et al. (2006) consists of 40 statements rated on a five-point Likert scale (e.g., item 7 “It does not make sense for an individual to worry about environmental degradation, since harmful effects are produced collectively”; item 21 “It does not make sense to blame the individual who breaks a rule when everyone else does the same”; item 28 “It is often inevitable to resort to force in order to protect one’s own interests”). The purpose is to evaluate the tendency to employ disengagement mechanisms in response to various transgressions encountered in daily life, across different contexts, interpersonal relationships, and moral codes. The DMC assesses several dimensions, including Moral Justification (MJ; items 16, 23, 28, 30, 37), Euphemistic Labeling (EL; items 1, 13, 17, 22, 40), Advantageous Comparison (AC; items 5, 15, 26, 29, 35), Displacement of Responsibility (DR; items 2, 6, 20, 25, 34), Spread of Responsibility (SR; items 7, 14, 21, 27, 38), Distortion of Consequences (DC; items 8, 10, 12, 19, 33), Attribution of Blame (AB; items 4, 11, 18, 24, 31), and Dehumanization of the Victim (DV; items 3, 9, 32, 36, 39). This scale will be utilized to assess divergent validity. High internal reliability was demonstrated in the validation sample (α = 0.91).

#### 3.2.4 Pro-environmental behaviors scale

The Pro-Environmental Behavior Scale (PEBS), designed by Markle (2013) and validated in Italian by Menardo et al. (2020) assesses individuals’ environmental attitudes, behaviors, and values, comprising 19 items (e.g., item 3: “How often do you limit your shower time to conserve water?”; item 4: “How often do you wait for a full load before using the dishwasher or washing machine?”; item 11: “In the past year, have you reduced your beef consumption?”). This scale will be utilized to evaluate predictive validity. The PEBS encompasses a wide spectrum of behaviors, categorized into four types: Conservation (CO; items 2, 3, 5, 6), Environmental Citizenship (EC; items 8, 9, 10, 11, 12, 13), Food (FO; items 14, 15, 16), and Transportation (TA; items 17, 18, 19). These behaviors, identified by environmental scientists, are deemed to have significant consequences for the environment. The internal reliability demonstrated in the validation sample was satisfactory (α = 0.71).

### 3.3 Data analysis

Two distinct samples were recruited. The first, consisting of 146 participants, was used to conduct an Exploratory Factor Analysis (EFA), while the second, consisting of 161 participants, was used for a Confirmatory Factor Analysis (CFA) to validate the structure identified in the EFA.

#### 3.3.1 Exploratory analysis

To determine the dimensionality of the scale and identify any unsuitable items, we conducted an Exploratory Factor Analysis (EFA) using IBM SPSS Statistics version 27 and Jamovi statistical software version 2.5. A Principal Components Analysis (PCA) and Parallel Analysis (PA), as proposed by Horn (1965), were performed on the 10 items. Parallel Analysis adjusts for sampling error by comparing eigenvalues obtained from PCA with those derived from random data. Before conducting PCA, we assessed the adequacy of the correlation matrix for factor analysis using Bartlett’s test of sphericity and the Kaiser-Meyer-Olkin (KMO) test. A significant Bartlett’s test (*p* < 0.05) and a KMO index >0.70 indicate adequacy of the correlation matrix. PCA was carried out with oblique rotation (Promax) due to the presumed interrelatedness of factors. Items with a loading ≥0.30 (as suggested by Hair et al., 1998) on a single factor were retained for further analysis. The PCA solution was then validated using results from Parallel Analysis. Subsequently, we assessed the internal consistency of the questionnaire and identified problematic items (i.e., items whose removal improved Cronbach’s alpha). As no items were removed and the version with all 10 items demonstrated excellent reliability (α > 0.95), it was selected for further analysis.

#### 3.3.2 Confirmatory analysis

Confirmatory Factor Analysis (CFA) was used to assess the factorial validity of the DEN, considering the model proposed by Greenberg et al. (2017). This analysis, conducted using Jamovi statistical software version 2.5, aimed to examine the internal validity of the DEN by analyzing its items. Additionally, Multi-group CFA was carried out using JASP Team (2020) to test whether the same number of factors is extracted between groups. To evaluate the goodness-of-fit of the factor structure, the χ^2^/df ratio was examined, with a value of ≤3 considered acceptable. Hu and Bentler’s (1999) guidelines for fit indices were also employed. These included: (a) Comparative Fit Index (CFI), with values ≥0.90 indicating a good fit; (b) Tucker Lewis Index (TLI), with values ≥0.90 indicating a reasonable fit; (c) Root Mean Square Error of Approximation (RMSEA), with values between 0.05 and 0.08 indicating adequacy of the model, and values ≤0.05 indicating evidence of absolute fit; (d) Standardized Root Mean Square Residual (SRMR), with values ≤0.08 indicating an adequate fit. The CFA yielded favorable results across the aforementioned indices, indicating a good fit of the model. Furthermore, multigroup CFA was conducted to examine whether the same number of factors was extracted across different groups. This technique is essential for testing the measurement model invariance across groups, ensuring that the construct is measured similarly in all groups. It allows for valid comparisons between groups, reducing measurement bias and improving construct validity. Additionally, multigroup CFA supports the generalizability of results to different populations or contexts.

#### 3.3.3 Correlations

The validity of the DEN was evaluated by correlating its factors with theoretically related measures. Firstly, Pearson correlation coefficients (*r*) were computed between the DEN factors and the CNS to establish convergent validity. Secondly, correlations were repeated between the DEN and the DMC scale to examine divergent validity. Lastly, correlations were conducted between the DEN and PEBS to assess predictive validity. In interpreting the correlations, Cohen’s guidelines (1988) were followed to assess effect sizes.

## 4 Results

### 4.1 Exploratory factor analyses

**Figure 1:**
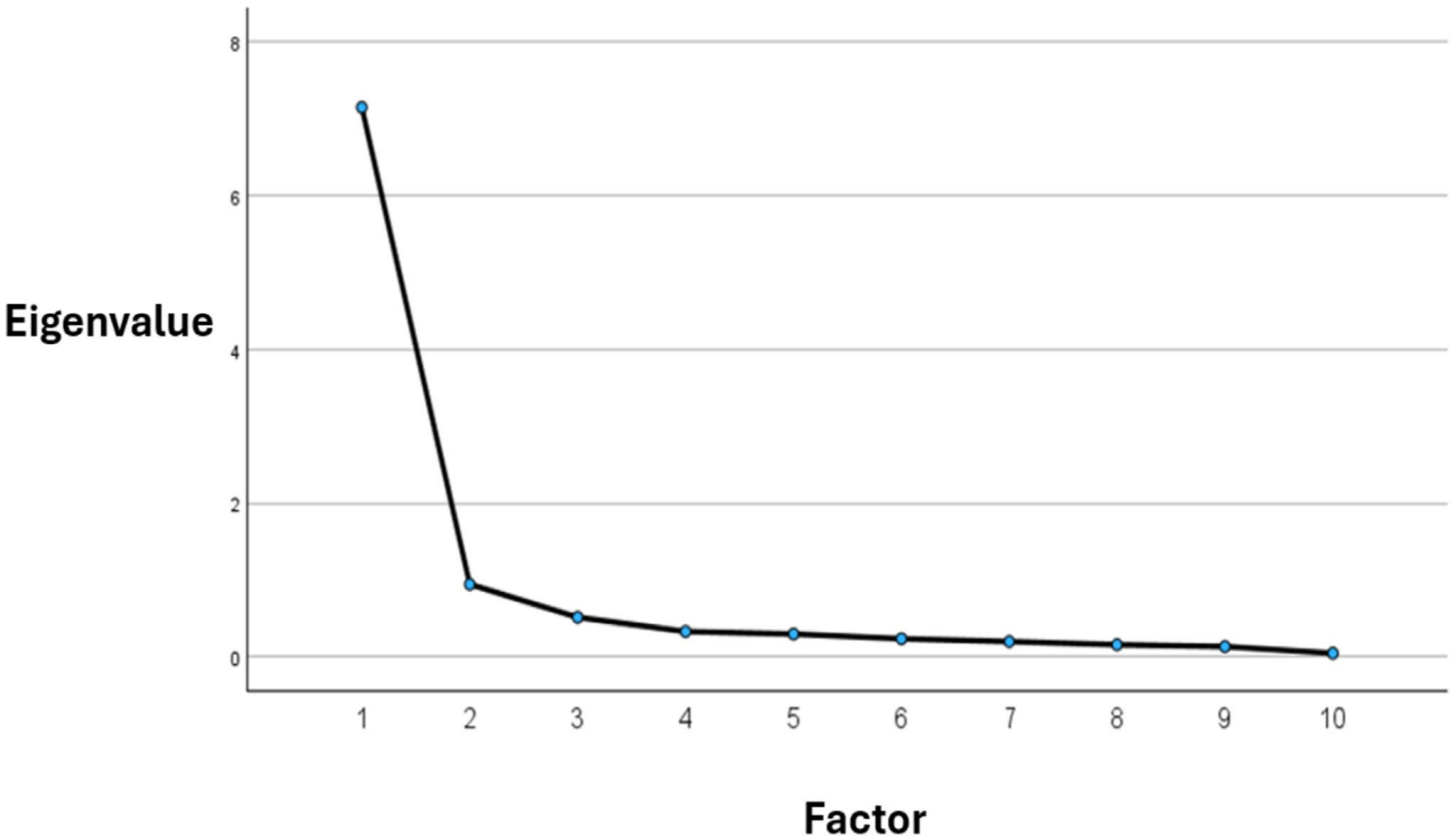
Scree plot, eigenvalues for factor analysis.

We performed both descriptive item analysis and EFA on a sample drawn from the general population. Our aim was to establish the mono-factorial structure of DEN, in line with the framework suggested by Tam (2013). A PCA was utilized to explore the factor structure of the 10 items. The correlation matrix was suitable for factor analysis, as evidenced by Bartlett’s test of sphericity (χ^2^ = 1563.232, df = 45, *p* = 0.001) and the Kaiser-Meyer-Olkin (KMO) measure of sampling adequacy (KMO = 0.906). The PCA revealed a one-factor structure. To confirm this structure, Parallel Analysis (PA) was conducted on DEN data, which is considered the most accurate method for component extraction. The results of the analysis confirmed a single component, thereby validating the structure obtained from the PCA. Additionally, examination of the scree plot (Figure 1) further supported the suitability of the one-factor solution.

### 4.2 Reliability

The DEN had excellent internal consistency, with a Cronbach alpha coefficient of 0.955. We also calculated McDonald’s Omega to provide a more robust estimate of internal consistency, as it accounts for varying factor loadings among items. Including Omega alongside other reliability metrics ensures a more comprehensive validation of the scale, aligning with best practices in psychometric evaluations (Dunn et al., 2014; McDonald, 1999). Our analysis revealed an Omega coefficient of 0.957, indicating excellent reliability of the scale. This high value reflects the strong internal consistency and supports the scale’s robustness for measuring the intended construct.

No significant changes were observed from the original version of the scale in terms of reliability. Additionally, none of the items were removed from the scale.

### 4.3 Confirmatory factor analysis

**Figure 2:**
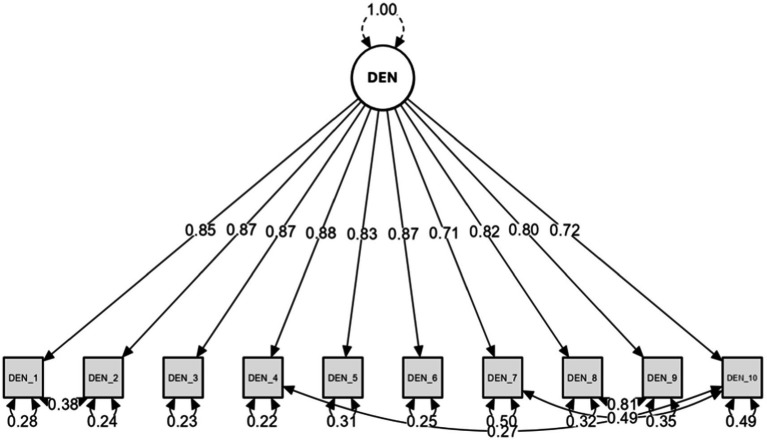
Graphical summary of the CFA.

CFA was conducted on the one-factor model. Bartlett’s test of sphericity confirmed the correlation between the items (χ^2^ = 59.253, df = 31, *p* = 0.002), while the Kaiser-Meyer-Olkin (KMO) measure indicated sample adequacy (KMO = 0.925). The factorial solution showed a good fit to the data (χ^2^/df = 1.911; CFI = 0.983; TLI = 0.976; SRMR = 0.031; RMSEA = 0.075 [CI] = 0.045–0.104). Although most of the indices met the recommended cut-off values (SRMR = 0.060; RMSEA = 0.070), opportunities for model improvement were identified through modification indices (MI) >10, indicating correlations between the errors of specific item pairs. Thus, items 8 and 9 (MI = 126.986), 7 and 10 (MI = 40.602), 1 and 2 (MI = 22.376) and 4 and 10 (MI = 14.366) were correlated. The CFA was re-run with these adjustments, resulting in a satisfactory fit of the factorial model (see Table 2; Figure 2). In addition, a multigroup CFA confirmed the model’s robustness across genders. Specifically, configural invariance demonstrated a consistent factor structure, metric invariance showed equivalent factor loadings, and scalar invariance indicated consistent item intercepts. These findings validate the model’s applicability and allow for meaningful comparisons between male and female participants (see Table 3 for detailed results and fit indices).

**Table 2:** Fit indices of empathy with nature scale generated by CFA.

Index	Index cut off	Value
*X*^2^/df	≤3	1.91
Comparative Fit Index (CFI)	≤90	0.983
Tucker-Lewis Index (TLI)	≤90	0.976
Root Mean Square Error of Approximation (RMSEA)	0.05–0.08	0.075
RMSEA 90% CI lower bound	0.05	0.045
RMSEA 90% CI upper bound	0.08	0.104
Standardized Root Mean Square Residual (SRMR)	≤0.08	0.031

**Table 3:** Godness-of-fit indices generated by the multigroup CFA cross gender.

	Index cut off	Value configural	Value metric	Value scalar	Value strict
*X*^2^/df	≤3	1.516	1.32	1.33	1.45
Comparative Fit Index (CFI)	≥0.90	0.981	0.987	0.985	0.975
Tucker-Lewis Index (TLI)	≥0.90	0.972	0.983	0.982	0.976
Root Mean Square Error of Approximation (RMSEA)	0.05 0.08	0.080	0.064	0.064	0.075
Standardized Root Mean Square Residual (SRMR)	≤0.08	0.035	0.045	0.050	0.055

The adequacy of the dataset for factor analysis was confirmed using Bartlett’s test of sphericity. Bartlett’s test indicated a significant correlation among the items (χ^2^ = 133.592, df = 92, *p* = 0.003), CFI = 0.975, TLI = 0.976 SRMR = 0.055, RMSEA = 0.075, 90% CI = 0.045–0.102. While most fit indices met the recommended thresholds (e.g., SRMR ≤ 0.060, RMSEA ≤ 0.070), modification indices (MI > 10) suggested correlations between the errors of specific item pairs. These included items 8 and 9 (MI = 70.391), items 1 and 2 (MI = 17.069), and items 7 and 10 (MI = 10.705). To improve the model, these error correlations were accounted for, and the CFA was re-run. After adjustments, the model achieved a satisfactory fit, providing strong support for the proposed factorial structure (see Table 3).

### 4.4 Correlations

#### 4.4.1 Convergent validity

The DEN factor showed a significant correlation with the CNS factor, *r* (CNS) = 0.576 *p* < 0.001, CI 95% [0.463, 0.671]. Thus, the DEN was strongly correlated with CNS, configuring the DEN as a questionnaire assessing an individual feeling close to and in empathy with nature.

#### 4.4.2 Divergent validity

The DEN factor demonstrated an inverse correlation with the DMC. Specifically, the correlations were as follows: *r* (MJ) = −0.137, *p* < 0.001, 95% CI [−0.286, 0.018]; *r* (EL) = 0.039, *p* = 0.624, 95% CI [−0.117, 0.192]; *r* (AC) = 0.044, *p* = 0.577, CI [−0.111, 0.198]; *r* (DR) = − 0.143, *p* = 0.062, 95% CI [−0.292, 0.012]; *r* (SR) = − 0.147, *p* = 0.062, 95% CI [−0.292, 0.007]; *r* (DC) = −0.003, *p* = 0.971, 95% CI [−0.157, 0.152]; *r* (AB) = −0.018, *p* = 0.822, 95% CI [−0.172, 0.132]; *r* (DV) = −0.060, *p* = 0.451, 95% CI [−0.212, 0.096]. Moreover, the correlation between the total score of the DMC and DEN is not significant, *r* = 0.67, *p =* 0.40, 95% CI [−0.219, 0.089]. These results indicate that the DEN was negatively correlated with some dimensions of the DMC, suggesting that individuals who exhibit empathy toward nature tend to possess a strong civic sense.

#### 4.4.3 Predictive validity

The DEN correlated significantly with the PEBS factors, *r* (CO) = 0.087, *p* < 0.001 95% CI [−0.069, 0.238]; *r* (EC) = 0.312, *p* < 0.001, 95% CI [0.166, 0.445]; *r* (FO) = 0.202, *p* < 0.001 95% CI [0.048, 0.345]; *r* (FTA) = 0.062, *p* < 0.015, 95% CI [−0.093, 0.215]. Moreover, the correlation between the total score of the PEBS and DEN is significant *r* = 0.228, *p* < 0.04, 95% CI [0.076, 0.370]. Thus, the DEN was positively correlated with all the subscales of the PEBS as well as with the total score. This indicates that the DEN questionnaire effectively measures the relationship between empathy with nature and pro-environmental behavior.

## 5 Discussion

The aim of this study was to validate in the Italian context a questionnaire that measured empathy toward nature, Dispositional Empathy with Nature (DEN; Tam, 2013). We investigated the psychometric properties and convergent, divergent and predictive validity of DEN. The results reported in this article provide good evidence that the DEN is a reliable and valid scale.

We proceeded with an EFA, followed by a subsequent CFA, as is frequently observed that scales translated into different languages and examined within diverse cultural frameworks may not exhibit the same underlying factor structure as the original version. As with the original version, it has been shown that the elements that compose the scale load on a single factor and show a high internal consistency. Following the CFA, it was determined that no items required to be deleted in order to strengthen the questionnaire’s structure. The original scale configuration remained the same, supported by adequate goodness-of-fit indices and strong factorial loadings. The model revealed the one factor structure associated with empathy toward nature, aligning with the structure observed in Tam’s original version (Tam, 2013). This highlights the robust structure of the scale, confirming its capacity to effectively assess individuals’ dispositional tendency to understand and empathize with the emotional aspects of the natural environment, even after its translation into Italian.

The strong correlations observed between the DEN and CNS (Mayer and Frantz, 2004) suggest a significant relationship between the individual’s connection to nature and their capacity for both emotional and cognitive empathy toward it (Mayer and Frantz, 2004; Tam, 2013; Nilsson et al., 2019). This suggests that those who report a deep bond with the natural environment are likely to also experience a heightened ability to understand and emotionally resonate with nature’s well-being (Zylstra et al., 2014; Tam, 2022). These results are in line with the theories of environmental psychology, according to which interconnection with nature is not just a passive affinity, but an active engagement that fosters cognitive empathy (i.e., understanding the needs and processes of nature) and emotional empathy (i.e., concern for the state of nature) (Chen et al., 2024). This suggests that the psychological construct of nature connectedness involves a multidimensional relationship, where emotional affinity enhances cognitive awareness, and vice versa. In practical terms, individuals with high nature connectedness may be more likely to engage in pro-environmental behaviors, driven by both an intellectual understanding of environmental issues and an emotional investment in nature’s health (Loy et al., 2024). These results provide valuable insights for environmental education and conservation efforts, where promoting a stronger emotional and cognitive connection with nature could lead to greater ecological protection.

Some correlations were identified among the DEN and some dimensions of the DMC. Those who scored lower in civic moral engagement also displayed decreased perceptions of responsibility toward the natural environment, resulting in a lower tendency to empathize with it. These results are in accordance with the idea that a person with low empathy toward the natural world will consequently engage less in behaviors to protect it (Kesebir and Kesebir, 2017). Some negative correlations were identified between specific DEN and DMC dimensions. Those who scored lower in civic moral engagement also showed a lower perception of responsibility toward the natural environment, resulting in a lower tendency to feel empathy for it. These results are consistent with the idea that a person with low empathy toward the natural world will consequently engage in less behaviors to protect it (Kesebir and Kesebir, 2017). However, literature (Bandura, 2002; Haddock and Jimerson, 2017; Hardy, 2017; Weisz and Cikara, 2020; Falla et al., 2021) suggests that, sometimes, moral disengagement behaviors are adopted by people as cognitive strategies to manage the complexities of environmental behavior. People may feel empathy for nature while using strategies like blame attribution or euphemistic labeling to cope with internal conflicts between their behaviors and environmental beliefs. For example, moral disengagement (as studied in bullying and aggression) involves cognitive mechanisms like minimizing responsibility or distorting consequences to justify actions that contradict personal values (Falla et al., 2021). Similarly, individuals with empathy for nature may still engage in strategies like blaming others for environmental harm or downplaying their role, helping them resolve the tension between their values and actions.

The Civilian Morale Disengagement construct is strongly related to the concept of morality as both are based on the idea that moral behavior depends not only on moral reasoning (“What is right to do in this situation?”), but also on positive connection (e.g., interest, empathy, pity, love) toward others, which then leads to a sense of responsibility (Sevillano et al., 2007; Schroeder et al., 2015; Young et al., 2018; Jing et al., 2022). For this reason, it becomes important to work on responsibility to nature. Promoting a sense of responsibility and embracing accountable behavior toward nature serves as a proactive measure, proving far more economical than remedying the repercussions of human overexploitation and interference in the natural world (Torii, 2015). Cultivating a sense of responsibility toward nature is an essential aspect of development that should be fostered from an early age. It is crucial for children to learn how to address complex issues, use interdisciplinary approaches, and evaluate facts and circumstances contributing to environmental degradation. Tailoring educational strategies to adolescents is an effective method for ecologically educating children. Nevertheless, education alone cannot foster ecological awareness without establishing a new ethical framework, such as ecological ethics, founded on respect for humanity and moral responsibility toward nature (Frankel-Goldwater, 2022). Based on these considerations, it is important to change people’s perception of moral commitment and responsibility to the environment to increase environmental protection.

The DEN scores exhibited a significant positive correlation with the subdimensions included in the PEBS scale as well as with the total score of the PEBS. Studies proposed a direct link between empathy toward nature and individuals’ environmental commitment and behavior, highlighting a relationship between nature, empathy, and pro-environmental actions (Wang et al., 2022). According to a study recently published by Blythe et al. (2021), the correlation between empathic engagement and sustainable actions could be leveraged to enhance the effectiveness of campaigns promoting environmental protection practices, while optimizing the political and economic resources used in their planning (Czap et al., 2018). In fact, a relationship has been identified between empathic involvement with nature and sustainable actions, highlighting how empathy for nature can have a double effect: on the one hand, by involving people in actions that aim to enhance the best environmental practices, and on the other hand, enabling people to optimize the relevant political and economic resources for planning them (Czap et al., 2018). Taking a broader view, this research proposes that environmental degradation could trigger emotional empathy and cognitive shifts, prompting individuals to take proactive measures to preserve the environment (Olivos and Clayton, 2017; Ives et al., 2018). However, this is not enough to resolve the climate crisis, and further research should focus on the role of environmental education. Ensuring public awareness and disseminating accurate information about environmental issues and conservation initiatives are critical. Providing individuals with precise knowledge about the environment can enhance their understanding of their negative actions, thereby fostering a change in their behavior (Sunassee et al., 2021). In the context of the ongoing global climate crisis, there is a strong demand for educational programs that integrate effective environmental strategies that can be implemented efficiently. Exploring students’ relationship with nature, the influence of their individual beliefs and behaviors, and the determinants that shape their actions can provide valuable insights to nurture substantial contributions to environmental conservation. Drawing on diverse disciplinary perspectives, environmental education seeks to translate human behavior into environmentally informed actions and to promote responsible environmental decision-making. By addressing these issues, environmental education has the potential to play a central role in addressing the world’s major ecological challenges (Sunassee et al., 2021). Encouraging students’ empathy for nature through environmental education in schools can increase their engagement in environmental conservation, thus promoting pro-environmental behaviors (Yusliza et al., 2020). Similarly, Larijani (2010) suggests that education is the most effective means of addressing environmental challenges, highlighting the crucial role of educational institutions in fostering greater environmental awareness through targeted environmental education (Larijani, 2010). Thus, it can be argued that as individuals cultivate empathy toward nature, they are increasingly inclined to prioritize environmental commitment and engage in pro-environmental actions (Wang et al., 2022).

## 6 Conclusion and limitations

The DEN demonstrated good psychometric properties, like Tam’s original version (Tam, 2013). In addition, correlations supported the usefulness of the DEN as a valid psychological tool for exploring emotional connections with nature. Overall, our study provided evidence for the internal consistency of the scale and its convergent, divergent, and predictive validity in the Italian context. However, it is important to interpret our results considering some limitations of the study. In particular, the recruitment of participants was based on an online survey, potentially excluding people with limited Internet access or technological competence. However, replication studies with larger samples could support the results we obtained.

As last limitation of our study, we did not assess test–retest reliability by having participants complete the scale at two different time points (T0 and T1). We acknowledge that including such an analysis could have provided additional insights into the stability of the scale over time.

Tam (2013) described DEN as an indicator of an individual’s ability to connect emotionally with the natural world. This measure of empathy toward nature could prove to be a significant predictor of pro-environmental attitudes, beliefs and behaviors (Perrin and Benassi, 2009).

Climate change stands as one of the most significant crises humanities has ever encountered, with wide-ranging impacts on biodiversity, environmental justice, human rights, mass migration, and public health, among other important areas (Sevillano et al., 2007; Ardoin et al., 2020). As environmental challenges grow in urgency, they present obstacles to sustainable human development and have rightly become a central focus of global attention. People’s connection with nature profoundly influences their behaviors toward the environment. As the world faces increasing environmental threats, understanding more about and strengthening the connection between people and nature is the key to stimulating actions to protect the environment. When people are aware about the benefits that individuals and communities receive from nature, they are much more likely to protect it from actions designed to destroy it (White et al., 2017; Ronen and Kerret, 2020). In this light, future research may explore the relationship between the DEN and environmental education that, among various aims, deal with the development of pro-environmental practices for the well-being of the environment (Ardoin et al., 2020). Moreover, empathy toward the environment and the earth is strongly linked to the concept of care. Both are based on the idea that when we empathize, and consequently care for something or someone outside of ourselves, we also cherish the world (Stuart-Smith, 2020). The emotional importance of repair tends to be overlooked in the world we live in today, but it plays a crucial role in our mental health. Consequently, another possible future study could be to investigate the relationship between empathy, nature and the environment. Moreover, it could be necessary to further explore the internal mechanism of how empathy with nature affects an individual’s pro-environment behavior (Gilli et al., 2022).

## Data Availability

The raw data supporting the conclusions of this article will be made available by the authors, without undue reservation.
